# Opposite effects of tissue inhibitor of metalloproteinases-1 (TIMP-1) over-expression and knockdown on colorectal liver metastases

**DOI:** 10.1186/1756-0500-5-14

**Published:** 2012-01-09

**Authors:** Obul R Bandapalli, Eva Paul, Peter Schirmacher, Karsten Brand

**Affiliations:** 1 Institute of Pathology, University Hospital Heidelberg, University of Heidelberg, Im Neuenheimer Feld 220/1, 69120 Heidelberg, Germany

## Abstract

**Background:**

Tissue inhibitors of metalloproteinases (TIMPs) and the corresponding metalloproteinases are integral parts of the protease network and have been shown to be involved in cancer development and metastasis. Paradoxically, for TIMP-1, tumor promoting as well as tumor inhibitory effects have been observed.

**Methods:**

To address this paradox, we utilized the BALB/c/CT26 mouse model that reliably leads to liver metastasis after splenic tumor cell injection and variegated the type of target cells for therapeutic intervention and the modalities of gene transfer. Since we have observed before that over-expression of TIMP-1 in liver host cells leads to efficient tumor growth inhibition in this model, we now examined whether targeting the tumor cells themselves will have a similar effect.

**Results:**

In concordance with the earlier results, TIMP-1 over-expression in tumor cells led to a dramatic reduction of tumor growth as well. To evaluate any influence of treatment modality, we further examined whether TIMP-1 knockdown in the same animal model would have the opposite effect on tumor growth than TIMP-1 over-expression. Indeed, TIMP-1 knockdown led to a marked increase in tumor burden.

**Conclusion:**

These data indicate that in the BALB/c/CT26 model, the modification of TIMP-1 has concordant effects irrespective of the type of target cell or the technique of modulation of TIMP-1 activity, and that TIMP-1 is unequivocally tumor inhibitory in this model.

## Background

Colorectal carcinoma is the second most frequent cancer disease in both sexes [1]. For patients with this type of cancer, liver metastases are the main cause of death. They often remain the only manifestation of the disease once the primary tumor has been surgically removed [2,3].

Therefore, successful treatment of liver metastases has the potential to cure the patient, and thus this is an area under intensive investigation. Besides standard treatment modalities like surgical intervention and chemotherapy, a number of molecular-based approaches for the treatment of colorectal liver metastases have been examined during the last 2 decades [4]. Among those, a modulation of the cancer cells' microenvironment has gained increasing interest [5-7], because a permissive host environment at the primary invasive site as well as at the site of metastasis is a prerequisite for successful tumor cell invasion.

Which are the potential target molecules? Excessive degradation and remodeling of the extracellular matrix (ECM) is one of the hallmarks of cancer progression at nearly every step of the metastatic cascade. Proteases contribute to each step from the first breakdown of the basal membrane of the primary tumor up to the extended growth of established metastases [8]. Among others, matrix metalloproteinases (MMPs) are a family of 24 enzymes that play an important role in this process. Naturally occurring tissue inhibitors of metalloproteinases (TIMPs 1 to 4) normally regulate and counterbalance the proteolytic activity of MMPs by binding to both the latent and active forms of MMPs in a 1:1 stoichiometry [9,10]. Consequently, over-expression of TIMPs by means of gene transfer [11-13] as well as by application of synthetic MMP inhibitors [13] has shown marked antitumor activity in various animal models. However, contradictory tumor growth promoting effects of TIMPs 1 to 3 have been reported in vitro, which occasionally translated into promotion of cell growth and metastasis in vivo (for review [13]). In addition, clinical trials using synthetic MMP inhibitors were of limited success [14]. These conflicting data indicate that a deeper understanding of the MMP/TIMP interplay and of potential additional functions is required. It appears as if only a fairly comprehensive understanding of the proteolytic network will finally allow the development of MMP specific inhibitors and will allow for the judgment of which situations they can be applied to (for review [15]).

To contribute another brick to the protease network and to help clarify conflicting data, we have utilized the BALB/c/CT26 animal model for colorectal liver metastases that exhibits reliable and reproducible liver metastases upon splenic injection of tumor cells [12,16]. In this study, we examined by means of gene transfer whether the type of target cell or the modality of gene expression modification will have an effect on TIMP-1's putative growth-promoting or -inhibitory function. Our data indicate that TIMP-1 in our model exhibits unequivocal effects, because it is tumor protective irrespective of whether host cells or tumor cells are targeted and irrespective of whether knockdown technology or over-expression technology is used.

## Methods

### Cell lines and animal experiments

CT26 human colon adenocarcinoma cells were cultured in RPMI supplemented with 10% FCS, 2 mM glutamine, 100 IU/mL penicillin, and 50 mg/mL streptomycin. Liver metastases were induced in 6 to 8-week-old BALB/c mice by intrasplenic injection of tumor cells. Briefly, a small upper quadrant incision was used to expose the spleen, and 1 × 10^6 ^cells in 50 μl were injected into the lower splenic pole with a 30.5 gauge needle. The spleen was returned to the abdominal cavity, the peritoneum was closed by suture, and the skin with wound clips. Two weeks after tumor cell inoculation, the animals were sacrificed, and total liver weights were determined. Animal experiments were performed according to official guidelines, with permission (by regional board Karlsruhe) under File No. 35-9185.81/G-50/05.

### Tissue preparation, laser microdissection (LMD) and microarray hybridization

Frozen tissue blocks were cut into 15 μm sections using a cryostat (Leica, Wetzlar, Germany) and stained using cresyl violet, according to the Ambion LCM staining kit protocol (Austin, TX, USA). Four distinct cell populations were separately microdissected with LCM equipment (Molecular Machines & Industries, Eching, Germany; or PALM, Bernried, Germany): (a) pure liver tissue, at least 5 cm away from the invasive front; (b) liver invasive front tissue, extending up to 10 cell layers into the liver; (c) tumor invasive front tissue, extending up to 10 cell layers into the tumor; and (d) pure tumor tissue, at least 100 cell layers away from the invasive front. These compartments were arbitrarily selected due to prior experience and results from immunostaining of up-regulated genes (unpublished data). Microdissection was performed to yield sufficient material for microarray and qPCR analysis.

Total RNA from microdissected samples was extracted (RNeasy mini kit; Qiagen, Hilden, Germany), and quality was evaluated using an Agilent 2100 bioanalyzer (Waldbronn, Germany). For microarray analysis, 30 ng of RNA corresponding to 2500-3500 cells from each microdissected group were amplified (RiboAmp HS RNA amplification kit; Arcturus, Sunnyvale, CA, USA), labeled, and the resulting biotinylated cRNA targets were used to probe the murine genome MOE430 set (A + B) (Affymetrix, Santa Clara, CA). Hybridization was performed in duplicates. Altogether, 8 chips were hybridized (2 compartments × 2 sub-chips [A + B] × 2 [duplicates] = 8).

For estimation of the percentage of tumor tissue as compared to pre-existent liver parenchyma, whole livers were embedded in paraffin and hematoxylin/eosin stained according to standard procedures.

### Data analysis

Raw files (cel files) from the scanned images of the Affymetrix chips (run in duplicates) were normalized using Affymetrix GCOS software, and fold changes were calculated using Excel (Microsoft, Seattle, USA) software.

### Relative quantitative real time-PCR

Microdissection and RNA isolation for relative qPCR were essentially performed as for hybridization experiments; however, independent samples were used. Thirty nanograms of total RNA, corresponding to 2500-3500 cells were used for quantification. Reverse transcription, qPCR, normalization (on 18S RNA), and efficiency correction (on 18S RNA) were performed. Oligonucleotides for 18S RNA and IL-8 qPCR were designed using the Primer3 software (Whitehead Institute, Cambridge, MA, USA). The sequences for 18S RNA were as follows: forward primer, 5'-AAA CGG CTA CCA CAT CCA AG-3'; reverse primer, 5'-CCT CCA ATG GAT CCT CGT TA-3'. Primers for TIMP-1 were purchased (Cat No. QT00996282; QuantiTect^® ^primers, Qiagen GmbH, Hilden, Germany). All the experiments were done in triplicate and repeated twice.

### ShRNA-mediated down-regulation of TIMP-1

Cells were transfected with pSM2C plasmids containing 2 different TIMP-1 shRNAmir constructs (TIMP-1 ShRNA-317 and TIMP-1 ShRNA-234) (Open Biosystems, AL, Huntsville, USA) or a non-silencing shRNAmir construct, according to the manufacturer's instructions. Briefly, 2 × 10^5 ^CT-26 cells were seeded into 6 well plates and subsequently transferred using Arrest-In^™ ^transfection reagent (Open Biosystems) with the indicated shRNAmir (10 μg transfection solution containing 2 μg TIMP-1 shRNAmir or 2 μg control siRNA). After 6 h, the medium was replaced by standard culture medium. The cells were returned to the CO_2 _incubator at 37°C. Forty-eight hours later, the cells were grown in a complete medium supplemented with puromycin (2 μg/mL) for selection. Single clones were then selected from pooled populations by serial dilutions using standard protocols. Successful shRNA transfection was confirmed by qRT-PCR and ELISA. All the experiments were performed in triplicates and repeated twice.

### Over-expression of TIMP-1

Lentiviral TIMP-1 viruses (LvhuTIMP-1) were generated by co-transfection of pLenti6/V5-DESThuTIMP-1 vector containing human TIMP-1 under the control of the CMV promoter with the ViraPower^™ ^packaging plasmid mixture: pLP1, pLP2, and pLP/VSV-G (Invitrogen) into 293FT cells using Lipofectamine 2000 (Invitrogen). CT26 cells were infected with LvhuTIMP-1 viruses and transduced cells were selected by blasticidin (5 μg/mL).

### ELISA

Enzyme-linked immunosorbent assay (ELISA) was used to determine the concentration of TIMP-1 in TIMP-1 over-expressing and down-regulated cells compared to the wild type control cells and non-coding shRNA control cells using Quantikine human and mouse TIMP-1 assay kit according to the manufacturer's instructions (R&D systems, Wiesbaden, Germany). All the experiments were performed in triplicates and repeated twice.

### Statistical analysis and software

Data are presented as the mean ± standard deviation. The statistical comparison between groups of animal experiments was accomplished with the non-parametric Wilcoxon test.

## Results

### TIMP-1 is expressed in liver parenchyma and tumor compartments

**Figure 1:**
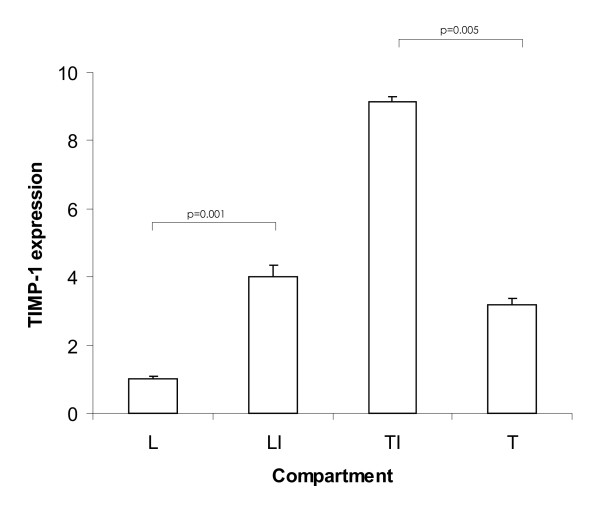
**Gene expression of TIMP-1 in 4 different compartments of mouse livers harboring colorectal liver metastases**. Bars represent fold changes of TIMP-1 mRNA with the values obtained in pure liver tissue set as baseline. L, liver tissue; LI, liver part of the invasion front; TI, tumor part of the invasion front; T, tumor tissue.

We first wanted to examine to what extent TIMP-1 is expressed in our animal model. In particular, we wanted to know, whether there is differential gene expression between different compartments of colorectal liver metastases, namely between the liver part of the invasion front (LI), liver away from the invasion front (L), tumor part of the invasion front (TI), and tumor away from the invasion front (T). As displayed in Figure 1, TIMP-1 mRNA levels in LI were 4-fold higher than in L and 2.9-fold higher in TI than in T. The ratio of pure tumor tissue to pure liver tissue was 3.2 to 1. The highest TIMP-1 levels were observed in TI, which were 9.1-fold higher than that in L. These data indicate that TIMP-1 is expressed in the tumor as well as in the liver, and that the invasion front compartments exhibit higher TIMP-1 levels than the inner parts of liver and tumor respectively.

### Construction of TIMP-1 knockdown and TIMP-1 over-expressing cells

**Figure 2:**
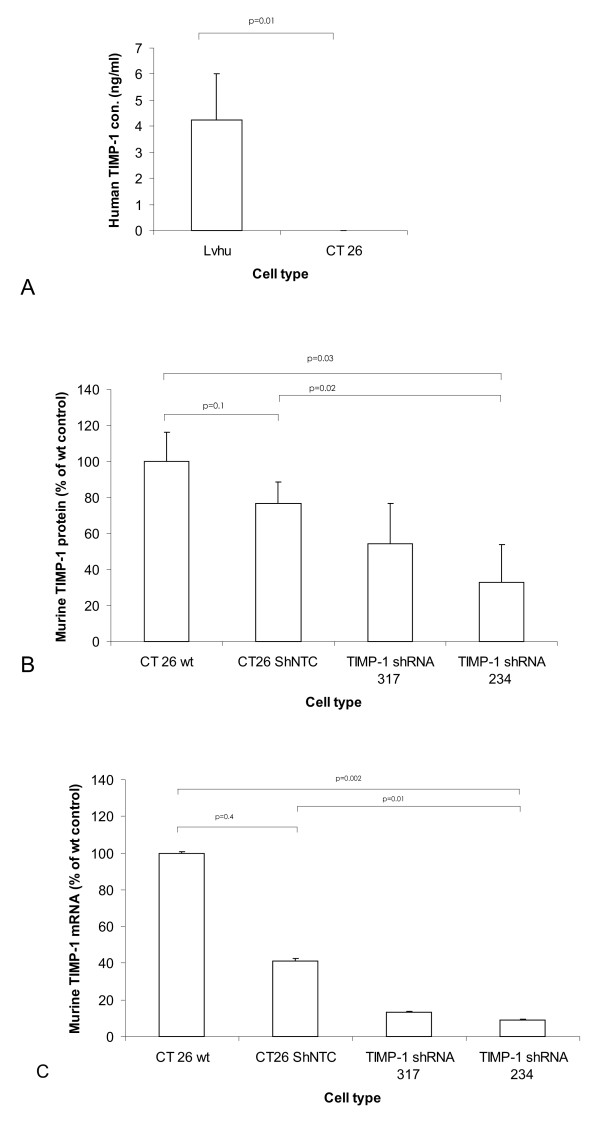
**Over-expression and knockdown of TIMP-1 in CT-26 murine colon carcinoma cells**.(**A**) Secretion of human TIMP-1 by stably TIMP-1-transduced cells (ELISA). (**B**, **C**) Secretion of murine TIMP-1 protein (B, ELISA) or expression of TIMP-1 mRNA (C, relative qPCR) by wild type cells or by stable clones harboring TIMP-1 shRNA or non-coding (NTC) shRNA.

In the next step, we constructed genetically modified cell lines to examine putative anti-metastatic TIMP-1 activity in vivo. We have earlier shown that adenoviral gene transfer of TIMP-1 [12] or TIMP-2 [11] into the unaffected liver tissue of mice can inhibit the growth of colorectal liver metastases. Since adenoviral gene transfer specifically targets the liver cells including hepatocytes, hepatic stellate cells, and macrophages, the observed antitumor effect was due to TIMP-1 expression by host cells. We now wanted to examine whether targeting of the tumor cells would lead to an antitumor effect as well. To this extent, CT26 cells were stably transduced with human TIMP-1 cDNA under the control of the CMV promoter. Production of human TIMP-1 by transduced CT26 cells was examined by ELISA (Figure 2A). Whereas no human TIMP-1 could be detected in wild type murine CT26 cells, a concentration of 4.2 ng/mL could be detected in the supernatants of TIMP-1 over-expressing cells.

In a second approach, we wanted to examine whether knockdown of endogenous TIMP-1 would lead to the opposite in vivo effect than over-expression of TIMP-1. To this extent, CT26 colon carcinoma cells were stably transduced with TIMP-1 shRNA or control shRNA, and TIMP-1 mRNA levels as well as protein levels were determined by semi-quantitative RT-PCR and ELISA, respectively. The best clone displayed a reduction of TIMP-1 mRNA by 90% (Figure 2C) and of TIMP-1 protein by 65% (Figure 2B). A fairly strong and apparently unspecific reduction of TIMP-1 mRNA and protein was observed if scrambled control RNA was used.

### TIMP-1 knockdown and TIMP-1 over-expression lead to opposite effects on metastatic tumor growth

**Figure 3:**
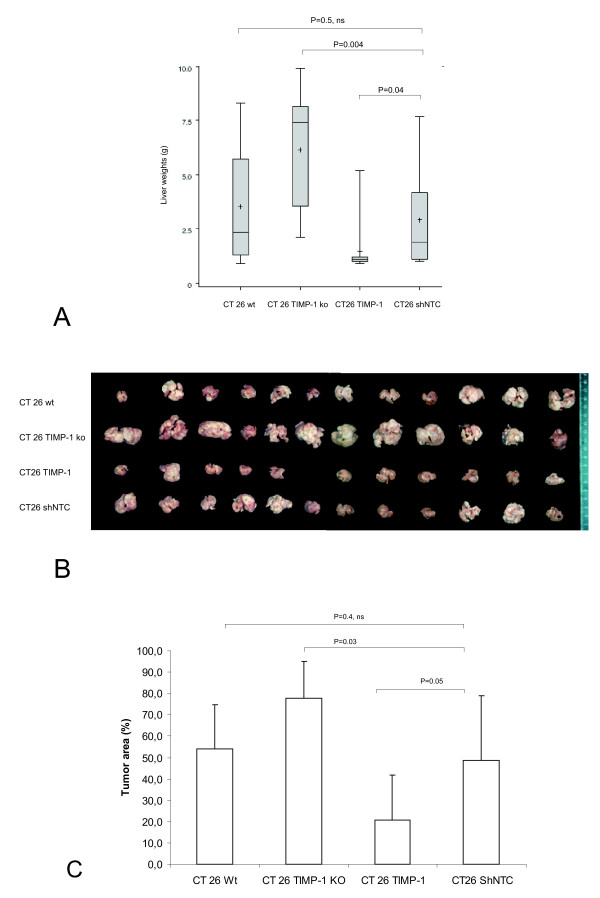
**Effect of over-expression and knockdown of TIMP-1 on tumor growth in vivo**. (**A**) Bars represent liver weights of BALB/c mice challenged with splenic injections of wild type and modified CT26 cells as indicated. The whole experiment was terminated, when the first animal had to be euthanized due to ethical guidelines. +: mean. (**B**) Photograph of resected livers. Any increase in liver weight is due to an increase in tumor burden. (**C**) Percentage of tumor tissue (area) as compared to pre-existent liver parenchyma.

To evaluate whether TIMP-1 over-expression by tumor cells will have an anti-metastatic effect like over-expression by stromal cells [12] and to evaluate whether knockdown of endogenous TIMP-1 will have the opposite effect, we applied the CT26 TIMP-1 over-expressing cells or CT26 TIMP-1 knockdown cells into the spleens of BALB/c mice and allowed liver metastases to develop. After 14 days, animals were sacrificed, and liver weights were determined. Over-expression of TIMP-1 led to a significant reduction of tumor burden, as compared to the wild type and the scrambled shRNA control groups (Figure 3A-C). In contrast, TIMP-1 knockdown increased tumor burden dramatically. Differences were significant with regard to both examined parameters: total liver weights (Figure 3A) and percentage of tumor as compared to pre-existent liver parenchyma (Figure 3C). No notable difference between wild type control cells and scrambled shRNA control cells was observed.

## Discussion and conclusions

In this study, we examined the effect of targeting tumor cells by modulation of TIMP-1 gene expression for the treatment of colorectal liver metastases. We observed a pronounced inhibition of tumor growth if TIMP-1 was over-expressed and a pronounced increase of tumor growth if TIMP-1 was knocked down.

The inhibition of tumor growth upon over-expression of TIMP-1 is concordant with our earlier observation in the same animal model of an inhibition of liver metastases if the liver host cells are targeted by adenoviral gene transfer [12]. In fact, the degree of tumor growth inhibition was even comparable as being roughly 2.5 to 4-fold in both studies. This result indicates that TIMP-1 exerts its metastasis inhibitory effect irrespective of whether the host microenvironmental cells or the tumor cells are targeted. Apparently, in both cases, sufficient amounts of TIMP-1 are secreted to act in a paracrine fashion to inhibit ECM-degrading MMPs. In addition, it appears as if the mere quantity of TIMP-1 expression is a decisive parameter to observe any in vivo effects. We have earlier observed that only high levels of TIMP-1 but not moderate levels expressed by host cells had an anti-tumor effect [12]. Similarly, in this current study, only a prominent reduction of TIMP-1 levels in the treatment group but not a moderate reduction in the NCT control group had any effect on tumor growth.

The antitumor effect of TIMP-1 is in agreement with a number of studies reporting decreased numbers and sizes of primary tumors and metastases upon TIMP-1 over-expression (for review [13]), including stomach cancer [17], melanoma [18], fibrosarcoma [19], and pancreatic cancer [20]). These effects argue in favor of a dominant antitumor effect of TIMP-1 in our model over any putative growth-promoting effects. In contrast, in a model of lymphoma and fibrosarcoma metastatic to the liver, moderate over-expression of TIMP-1 in transgenic mice did not change metastasis [21,22], whereas adenoviral gene transfer, which leads to very high levels of TIMP-1, had a pronounced anti-metastatic effect [12]. One reason for these conflicting data may be that only prominent modifications of TIMP-levels will have effects on tumor growth, whereas minor modifications can be compensated for by the tumor. Another reason may be profound inherent differences in the model systems.

Our studies on over-expression of TIMP-1 were performed with human TIMP-1. We were now interested to examine whether knockdown of endogenous murine TIMP-1 would have the expected opposite effect as over-expression of human TIMP-1. Indeed, the knockdown of endogenous murine TIMP-1 led to a dramatic and highly significant increase of tumor burden. To our knowledge, this is the first report of an in vivo effect of shRNA-mediated TIMP-1 knockdown. However, related studies underscore our findings: The knockdown of TIMP-1 in HeLa, C33A, and A549 cancer cells [23] as well as in corneal epithelial cells derived from pterygia [24] increased invasion and migration.

Since our gene expression studies revealed an increase of TIMP-1 gene expression in the invasion front we asked ourselves why further over-expression of TIMP-1 would lead to decreased tumor growth and why knockdown of TIMP-1 would lead to enhanced tumor growth as we observed? One explanation among others may be that a finely tuned balance of MMPs and TIMPs causing increased TIMP-1 in response to increased MMP is necessary in the active area of the invasion front, whereas in the inner parts of the tumor unrestricted MMP activity is more beneficial for rapid tumor growth.

In summary, our data indicate a fairly unequivocal anti-metastatic effect of TIMP-1 in our animal model. Any indications of an in vivo growth-promoting effect that has been reported before were not seen in our system. Targeting tumor cells appears to be at least as efficient as targeting the host microenvironment, and over-expression of human TIMP-1 has the opposite effect as knockdown of endogenous murine TIMP-1. Altogether, this study indicates that over-expression of TIMP-1 may be a treatment modality for colorectal liver metastases at least in selected situations.

## Competing interests

The authors declare that they have no competing interests.

## Authors' contributions

ORB performed the qPCR and microdissection experiments, contributed to the animal experiment and supervised the ELISA and cell culture experiments. EP performed the ELISA and cell culture experiments. PS provided the scientific environment and edited the manuscript. KB supervised the project, wrote the manuscript and contributed to the animal experiment. All authors read and approved the final manuscript.
